# Small watersheds may play a disproportionate role in arctic land-ocean fluxes

**DOI:** 10.1038/s41467-023-39209-7

**Published:** 2023-06-10

**Authors:** J. E. Vonk, N. J. Speetjens, A. E. Poste

**Affiliations:** 1 Department of Earth Sciences, Vrije Universiteit Amsterdam, Amsterdam, The Netherlands; 2 Norwegian Institute for Nature Research, Fram Centre for High North Research, Tromsø, Norway; 3 Norwegian Institute for Water Research, Fram Centre for High North Research, Tromsø, Norway

**Keywords:** Hydrology, Carbon cycle

## Abstract

While over 99% of coastal arctic rivers drain small catchments, future projections of land-ocean fluxes are based on data from large rivers. We encourage inclusion of and increased focus on smaller catchments to support representative assessments of arctic ecosystem change.

Inland waters integrate terrestrial, oceanic, and atmospheric reservoirs^1^ and contribute to the storage, processing, and emission of large amounts of terrestrial carbon. The arctic climate is warming three to four times faster than elsewhere on Earth^2^. Permafrost soils in the region, which store two atmospheres worth of carbon, have become vulnerable to thaw and decomposition^3^. Enhanced permafrost thaw, along with glacier mass loss and changing hydrological regimes, alter—and often increase—continental fluxes of freshwater and terrigenous material to the ocean^4,5^. Altered fluxes of organic matter, contaminants, nutrients, and sediments can impact the fishing, hunting, infrastructure, and subsistence of local communities, arctic coastal ecosystem functioning, and global climate feedbacks^3,6^.

While increased temperature and precipitation are occurring across the Arctic, the landscape response to these changes is far from homogeneous owing to differences in relief, ground ice content, soil properties, and presence of glaciers^7^. Despite their diversity a few apparent trends seem to emerge. There is a regime shift from snow-dominated to snow and rain-dominated systems, which causes an increase in fluvial transport during summer and early autumn^8^, when permafrost thaw and disturbance is at its maximum. Further, intensification of extreme summer rainfall increases the frequency and size of permafrost thaw slumps^9^, thaw depth^10^ and generally increases stream power and terrestrial-aquatic connectivity^8,11^. Smaller catchments in flat permafrost-dominated tundra, with polygonal landscape patterns caused by ice wedge formation^12^ (so-called ice wedge polygons; Fig. 1), are particularly sensitive to changes in summer precipitation and thaw depths^11^. In glaciated catchments, warming is driving increased land-ocean delivery of meltwater, inorganic sediments, and bedrock-derived material. The vastly different character of the northernmost small catchments along with the sharp hydrological shifts induced by climate change, causes fundamentally different and accelerated landscape-scale change here compared to larger river basins further south.

**Fig. 1:**
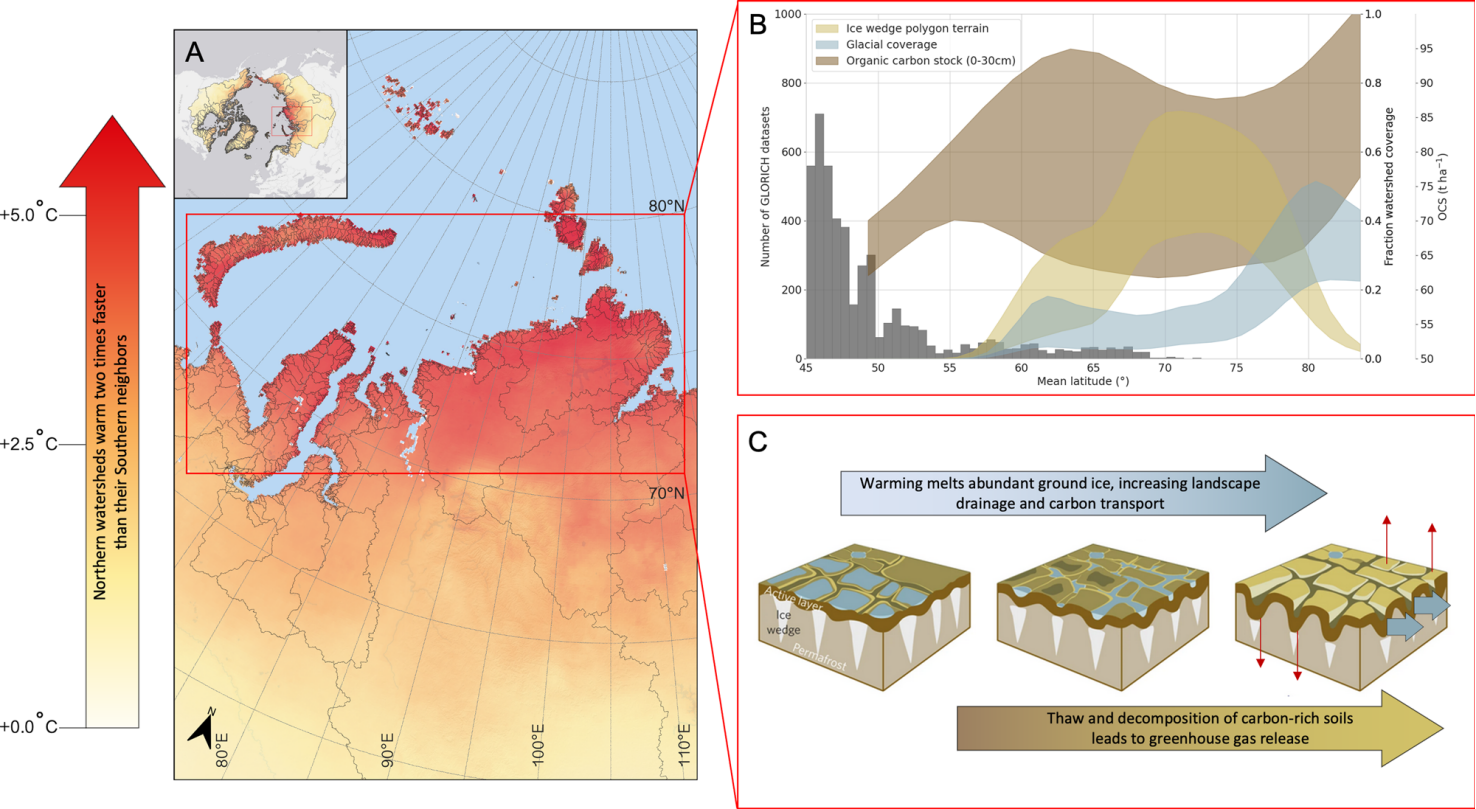
Coastal arctic watersheds. Nearly all northern catchments flowing into the Arctic Ocean (A) are smaller than 1000 km^2^. In these small catchments, climate warming occurs more than two times faster than the larger catchments to the south. Northern catchments (B) have high coverage of ice wedge polygon terrain, glacial coverage, and soil carbon stocks, and are often data poor^18^. Warming leads to (C) melting of ground ice and increased hydrological drainage and thaw and decomposition of carbon. Both processes increase transport and emission of carbon, accelerating greenhouse gas emissions. Polygon landscape images from ref. ^12^.

Coastal catchments smaller than 1000 km^2^ cover <10% of the entire pan-arctic watershed, and yet dominate land-ocean inputs along vast swathes of the arctic coastline^13^ (Fig. 1). These smaller coastal watersheds are highly abundant, experience rapid change, and have fundamentally different topography, climate, and geochemistry than the larger drainage basins. While we are beginning to understand the timing, magnitude, and geochemistry of lateral fluxes to the Arctic Ocean from the six largest arctic rivers^14^, known as the big six, we still know relatively little about the smaller northern catchments and what controls their fluxes of water and terrigenous input. Available studies of smaller watersheds e.g.^8,10,11,15^ suggest that simple extrapolation of large river fluxes likely underestimates total land-ocean export^16^. Such extrapolations introduce large uncertainties in climatic projections and in our understanding of current and future fluxes of carbon, nutrients, sediments and contaminants into the Arctic Ocean, and their roles in driving arctic marine ecosystem change.

The smaller, northern catchments are warming faster than their larger, more southernly neighbours, with +3.4 ± 1.8 °C warming between 1990 and 2019 in the north of Eurasia compared with +1.7 ± 0.65 °C further south^13^. Strong warming causes ground ice to melt which, in these flat coastal systems, sharply increases hydrological drainage and further thaw^12^. Spatial coverage of ice wedge polygon terrain also varies, with 38 ± 43% in the smaller northern catchments, versus just 2.0 ± 4.3% in the larger southern systems. At the same time, carbon stocks in the upper 30 cm of northern catchment soils are estimated to average 88 ± 13 tonnes/ha, compared to 67 ± 3.7 tonnes/ha for larger southern systems^13^. Soil carbon stocks between 30 and 200 cm are also higher for northern catchment soils, and these deeper stocks are still mostly contained within the permafrost. Warming could therefore turn these carbon sink landscapes into sources of carbon to the atmosphere, further complicating international efforts to keep the global temperature increase below 1.5 °C.

In order to understand the impact of warming and gain a realistic and representative view of the impact of this warming on the coupled pan-arctic terrestrial-aquatic carbon cycle, it is imperative that we move away from the dominant focus on catchment size. Although large catchments naturally integrate a multitude of smaller catchments, at the point where a large river meets the ocean all headwater stream variability is gone, as well as the possibility of obtaining a comprehensive assessment of the evasion and storage processes upstream in the watershed. Furthermore, the lack of knowledge on quantity and quality of fluxes from small coastal watersheds hampers our understanding of how these fluxes impact coastal ecosystem functioning, which is particularly important since these ecosystems support high biodiversity and are a source of food and livelihoods for coastal communities^6^. At present, a lack of long-term hydrological monitoring is the key limitation in understanding hydrology and lateral fluxes from smaller catchments across the pan-arctic.

We need to focus more attention on the smaller northernmost coastal watersheds. Increased efforts to monitor hydrology in these smaller arctic catchments is critical for estimating land-ocean fluxes, especially when paired with high-resolution sensor and sampling-based field monitoring^17^. To build process-understanding and an improved capacity for upscaling, field observations should be combined with hydrological and biogeochemical modelling of catchment behaviour and land surface models that include lateral fluxes. On regional scales, this process-understanding combined with available high-resolution remote sensing datasets, in-situ measurements and increasingly advanced machine learning algorithms could be turned into near-real-time predictions of lateral fluxes. These observational, monitoring, and experimental efforts must also be aggregated into a well-structured database so that they can become valuable assets to earth system modelers.

A new arctic is emerging, with a warmer, wetter, and less predictable climate. Pan-arctic projections and models use only large drainage basins, and coastal regions experiencing accelerated change are not included. It is time for us to shift our focus away from just the large arctic catchments and to instead act with synergised efforts to gauge the northern, coastal systems, in order to reliably upscale land-ocean fluxes and assess coastal arctic ecosystem change. These advances are needed for improving climate and earth system understanding and building adaption capacity for communities along the rapidly-changing pan-arctic coastline.
